# Associations between low muscle mass, blood-borne nutritional status and mental health in older patients

**DOI:** 10.1186/s40795-019-0330-7

**Published:** 2020-03-06

**Authors:** Salah Gariballa, Awad Alessa

**Affiliations:** 1 Internal Medicine, Faculty of Medicine & Health Sciences, United Arab Emirates University (UAEU), Al Ain, United Arab Emirates; 2 University of Sheffield, Sheffield, UK

**Keywords:** Muscle mass, Mental health, Older people, Acute illness

## Abstract

**Background:**

Although low muscle mass is an important predictor of increased physical morbidity in older patients, information on its impact on mental health and well-being is lacking. The first aim of this report is to look for associations if any between low muscle mass and mental health of older people in clinical practice. The second aim is to study underlying mechanisms including nutritional status.

**Methods:**

In this prospective longitudinal study we randomly selected and studied 432 hospitalized older patients’ baseline demographic data, clinical characteristics and nutritional status on admission, at 6 weeks and at 6 months. Low muscle mass was diagnosed using anthropometric measures based on the European Working Group criteria. Mental health outcome measures including cognitive state, depression symptoms and quality of life were also measured.

**Results:**

Out of 432 patients assessed 44 (10%) were diagnosed with low muscle mass. Patients diagnosed with low muscle mass at admission and over a 6-month follow up period had significantly poor cognitive function, quality of life and increased depression symptoms compared with those with normal muscle mass. After adjustment for poor prognostic indicators, age, disability, severity of acute illness and low muscle mass were associated with poor cognitive function and quality of life and higher depression symptoms in older patients over a 6 months period (*p* < 0.05). Although patients with low muscle mass had lower micronutrient concentrations compared to those patients with normal muscle mass, only serum albumin showed significant correlations with quality of life at admission and depression symptoms at 6 weeks.

**Conclusion:**

Low muscle mass is associated with poor blood-borne poor nutritional status and mental health in hospitalized older patients, however, this is partly explained by underlying co morbidity.

## Background

Despite growing evidence that low muscle mass is common and associated with increasing morbidity and mortality in older people, there is a lack of good-quality data of the effect of low muscle mass diagnosis on mental health and quality of life measures and their response to treatment [1–3]. Although a relationship between changes in body composition including muscle mass and mental well-being in older people has been postulated before, however supporting research evidence is weak [4]. Skeletal muscle cells are known to be metabolically active and secrete a number of substances that communicate directly with a number of organs including the brain and may therefore be responsible for the beneficial effects of muscle contraction/exercise on mental health [5]. Specifically postulated common underlying pathophysiological mechanisms linking changes in muscle mass with poor mental health and well-being include inflammation, oxidative stress, malnutrition and physical inactivity all are common and associated with ill health in older people [6]. A recent cross-sectional study on community –living older people has revealed that subjects with lower appendicular skeletal muscle mass had lower cognitive functioning scores than did participants with higher muscle mass scores [7]. We have recently reported that poor muscle strength in hospitalized older people is associated with poor physical and mental health [8]. A recent study however reported that poor muscle mass but not poor strength is associated with malnutrition in hospitalized older patients at admission [7]. Given the known link between malnutrition and poor mental health, the aim of this report is to present data on the relationship between low muscle mass, mental health and quality of life and underlying blood-borne nutritional status in hospitalized older patients.

## Methods

Subjects included in this research report were acutely ill-hospitalized older patients with common diagnoses who were part of for a randomized controlled trial [9] All subjects were admitted to Barnsley District General Hospital, UK. Barnsley District General Hospital serves a total population of 234,000. It has 650 beds and the medical unit has 250 beds for acute medical admissions. Patients were first, identified through the computerised hospital admission databases of all patients. When first admitted all patients have an individualised computerised plan created. The medical notes of those identified from the database were examined and eligible patients approached. Patients aged 65 years or older admitted with common diagnoses of ischaemic heart disease, heart failure, stroke, chest, urinary tract and blood infection, chronic obstructive lung disease, falls, syncope, and fractured limbs were included. Patients had to sign informed consent form to participate in the index study. Patient excluded from the study were those with severe medical or psychiatric illness including those living in institution. The study design was approved by Barnsley (UK) research ethics committee (REF: 04/Q2304/50). Data collected from medical records included demographic and clinical data admission diagnosis, previous illnesses, disability, medications, smoking and alcohol intake. All patients had three assessments the first within 72 h of admission in hospital and at 6 weeks and at 6 months either in hospital or in the community. Disability was assessed using the Barthel which scores 10 functions on a scale 0 (fully dependent) to 20 (independent). Standard anthropometric, hematological and biochemical data were used to assess nutritional status [9]. Method for determination of specific micronutrients concentrations has been published before [10]. Low muscle mass was diagnosed using The European Working Group on Sarcopenia in Older people (EWGSOP) criteria [2, 3]. The muscle mass was measured by mid-arm muscle circumference (MAMC) using the following formula: MAMC = MAC - (3.14 x triceps skinfold thickness). Low muscle mass was classified as MAMC less 21.1 cm and 19.2 cm in men and women respectively. Cognitive state was assessed by the abbreviated mental test questionnaire (AMT) [11]. The 15 item Geriatric Depression questionnaire (GDS) was used to measure depression symptoms [11]. The validated Medical Outcomes Study 36-items (SF-36) General Health Survey questionnaire used to assess quality of life [12].

### Statistical analysis

The independent student-t test or the nonparametric Mann-u-Whitney (SPSS version 24) were used to test differences in nutritional micronutrient variables between patients with low muscle mass and those patients with normal muscle mass. A *p*-value of < 0.05 is regarded as statistically significant. A forward stepwise multiple regression analysis was performed to determine the influence blood-borne nutritional markers on mental health outcome measures including cognitive state, depression symptoms and quality of life at admission and at 6 weeks. We also used multivariate analysis to determine the influence of low muscle mass on mental health and quality of life after adjusting for a number of independent poor prognostic clinical indicators including age, gender, disability, comorbidity (previous illnesses), and severity of acute illness (inflammation) measured using CRP.

## Results

**Fig. 1:**
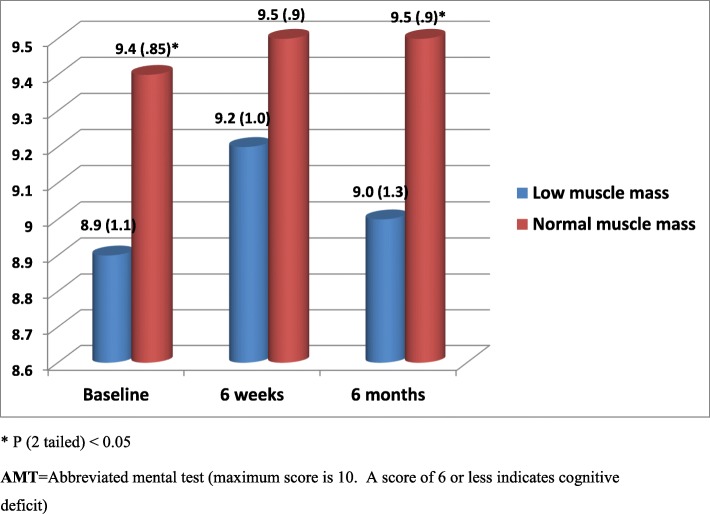
Cognitive state during both acute illness and recovery for study patients diagnosed with Low muscle mass compared with those normal muscle mass

**Fig. 2:**
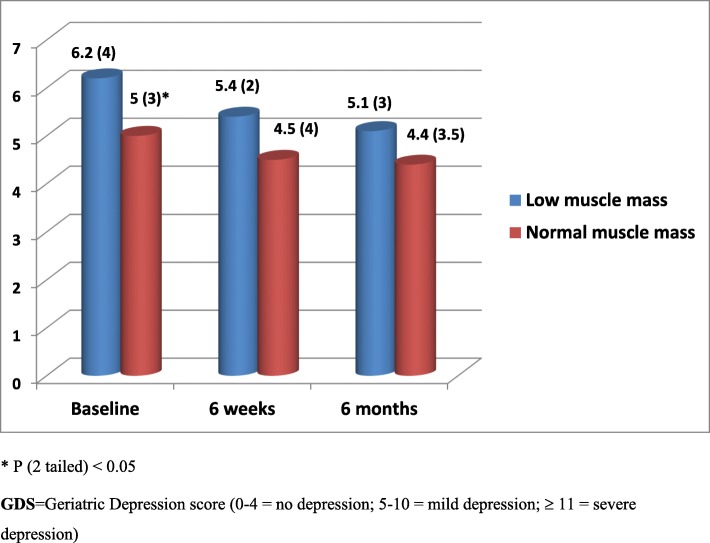
Depression symptoms during both acute illness and recovery for study patients diagnosed with low muscle mass compared with those with normal muscle mass

**Fig. 3:**
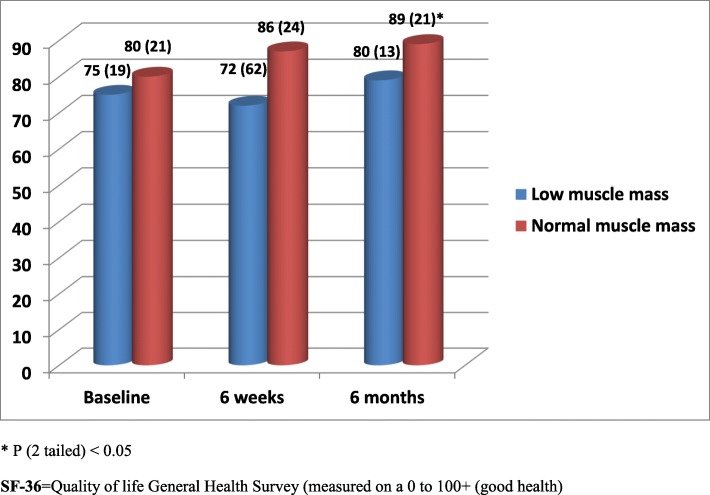
Quality of life during both acute illness and recovery for study patients diagnosed with Low muscle mass compared with those with normal muscle mass

All 432 acutely ill older patients aged 65 to 92 years [211 (49%) female] admitted to hospital and followed up for period of 6 months were included in this analysis. Among the 432 patients recruited 42 (10%) had low muscle mass at baseline. Corresponding figures at 6 weeks and at 6 months were 15 (10%) and 9 (6%). Exclusions were due to early discharge, death or inability to provide outcome data at follow up visits. Patients with low muscle mass at admission and at 6 weeks and 6 months follow up had significantly poor cognitive function, quality of life and/or increased depression symptoms compared with patients with normal muscle mass (Figs. 1, 2 and 3). Multiple regression analysis for the association between low muscle mass and other clinical indicators at admission revealed significant correlations between age, disability, low muscle mass and cognitive function (*p* < 0.05). Although tissue inflammation showed significant associations with both cognitive function and depression symptoms, chronic illness revealed significant and independent associations with cognitive function, depression symptoms and quality of life (Table 1). Table 2 shows nutritional biomarkers in patients with low muscle mass compared to those patients with normal muscle mass. The results show lower concentrations of nutritional biomarkers in patients with low muscle mass but the differences were only statistically significant for baseline serum albumin, plasma zinc and red cell folate only (*p* < 0.05). Ascorbic acid, zinc, lycopene, folate, and retinol were also significantly lower in patients with low muscle mass at 6 weeks (Table 2). We found no statistically significant associations between nutritional biomarker concentrations and mental health or quality of life scores except for serum albumin on quality of life at admission and depression symptoms at 6 weeks (Tables 3 and 4).

**Table 1:** Multiple regression result of age, gender, disability, co-morbidity, severity of acute illness and presence of low muscle mass on AMT, GDS and SF-36 on admission

	Abbreviated mental test score (AMT)		Geriatric Depression Score (GDS)		Quality of life Scores (SF-36)	
	Standardized Regression coefficient (95% C.I)	*P* value	Standardized Regression coefficient (95% C.I)	*P* value	Standardized Regression coefficient (95% C.I)	*P* value
Age (years)	−.12 (−.03 to −.003)	0.021*	−.01 (−.06 to .055)	0.880	.001 (−.36 to .37)	0.987
Gender	−.07 (−.30 to .05)	0.158	.001 (−.73 to .73)	0.992	−.027 (−5.6 to 3.3)	0.619
Barthel score	.12 (−.003 to .043)	0.025*	−.15 (−.20 to −.033)	0.006*	.12 (.03 to 1.04)	0.039*
Chronic illnesses	.002 (−.06 to .06)	0.962	.06 (−.11 to .40)	0.255	−.16 (−3.7 to −.74)	0.004*
CRP (mg/L) +	.11 (.00 to .003)	0.033*	−.18 (−.014 to −.004)	0.001*	.096 (−.004 to .061)	0.081
Low muscle mass (yes/no)	.12 (.05 to .60)	0.021*	−0.07(−.19 to .44)	0.224	.05 (−3.9 to 9.93)	0.387

* *P* value < 0.05 + CRP = C-reactive protein (measure of inflammation)

**Table 2:** Baseline & follow up biochemical nutritional and inflammatory markers for patients diagnosed with low muscle mass compared with those with normal muscle mass

Nutritional variable	Baseline mean (SD)		6 weeks		6 months	
	Low muscle mass (*n* = 42)	Normal muscle mass (*n* = 360)	Low muscle mass (*n* = 15)	Normal muscle mass (*n* = 154)	Low muscle mass (*n* = 9)	Normal muscle mass (*n* = 151)
C-reactive protein (mg/L)	51 (59)	52 (74)	7 (6)	12 (26)	13 (15)	11 (20)
Albumin (g/L) +	35.5 (5)	38.1 (5) *	39.6 (4)	40.8 (3)	40 (5)	41 (3)
Red cell folate (nmol/L)	144 (48)	195 (108) *	160 (45)	227 (117) *	187 (9)	215 (155)
Riboflavin, EGRAC+	1.279 (0.13)	1.310 (0.18)	1.278 (.15)	1.273 (.2)	1.250 (.2)	1.276 (.14)
Vitamin B_12_ (pmol/L)	377 (216)	390 (317)	506 (500)	361 (256)	444 (246)	340 (239)
Ascorbic acid (μmol/L)	17 (20)	24 (21)	18 (13)	35 (23) *	47 (56)	30 (26)
α-tocopherol (μmol/L)	25 (6)	25 (7)	29 (6)	27 (7)	29 (7)	26 (8)
Carotene (umol/L)	0.275 (.3)	.257 (.2)	.230 (.08)	.279 (.19)	272 (.17)	.262 (.15)
Retinol (umol/L)	1.16 (.5)	1.22 (.5)	1.69 (.5)	1.36 (.4) *	1.41 (.7)	1.36 (.5)
Lycopene (umol/L)	.121 (.09)	.158 (.1)	.108 (.06)	.154 (.1) *	.119 (.1)	.151 (.1)
Zinc (ng/ml)	571 (127)	629 (148) *	605 (110)	684 (150) *	665 (150)	644 (138)
Copper (ng/ml)	1110 (190)	1062 (230)	1120 (241)	1045 (246)	1036 (157)	956 (211)
Selenium (ng/ml)	63 (18)	68 (22)	68 (28)	83 (28)	84 (30)	74 (20)

* + *p* value < 0.05 for cumulative between (+) and within group (*) differences at baseline or at 6 weeks, + EGRAC = Erythrocyte Glutathione Reductase Activity Coefficient (EGRAC > 1.3 indicates riboflavin biochemical deficiency)

**Table 3:** Multiple regression result of blood-borne nutritional markers on AMT, GDS and SF-36 at admission

	Abbreviated mental test score (AMT)		Geriatric Depression Score (GDS)		Quality of life Scores (SF-36)	
	Standardized Regression coefficient (95% C.I)	*P* value	Standardized Regression coefficient (95% C.I)	*P* value	Standardized Regression coefficient (95% C.I)	*P* value
Albumin (g/L) +	.01 (−.04 to .05)	0.780	−.02 (−.28 to .05)	0.154	1.5 (.48 to 2.5)	0.005*
Red cell folate (nmol/L)	.001 (−.01 to .01)	0.527	−.001 (−.005 to .002)	0.395	−.003 (−.02 to .02)	0.756
Ascorbic acid (μmol/L)	.01 (−.006 to .017)	0.376	−.014 (−.06 to .03)	0.536	−.01(−.29 to .27)	0.940
Retinol (umol/L)	−.09 (−.39 to .22)	0.580	.24 (−.89 to 1.4)	0.674	−5.1 (−11.9 to 1.9)	0.151
Lycopene (umol/L)	.24 (−.94 to 1.42)	0.691	−3.9 (−8.5 to .75)	0.100	25 (−.81 to 51)	0.057
Zinc (ng/ml)	.001 (−.001 to .002)	0.724	−0.01 (−.19 to .004)	0.766	−.01 (−.04 to .02)	0.633

* *P* value < 0.05

**Table 4:** Multiple regression result of blood-borne nutritional markers on AMT, GDS and SF-36 at 6 weeks

	Abbreviated mental test score (AMT)		Geriatric Depression Score (GDS)		Quality of life Scores (SF-36)	
	Standardized Regression Coefficient (95% C.I)	*P* value	Standardized Regression coefficient (95% C.I)	*P* value	Standardized Regression coefficient (95%C.I)	*P* value
Albumin (g/L) +	.06 (−.08 to .12)	0.723	−.4 (−.87 to −.09)	0.017*	.29 (−.59 to 5.7)	0.109
Red cell folate (nmol/L)	−.27 (−.003 to .001)	0.065	.05 (−.01 to .008)	0.760	−.02 (−.06 to .05)	0.891
Ascorbic acid (μmol/L)	.05 (−.001 to .02)	0.743	−.02 (−.07 to .06)	0.866	- .06 (−.59 to .40)	0.706
Retinol (umol/L)	.09 (−.43 to .89)	0.490	.04 (−2.2 to 2.8)	0.805	−.09 (−25 to 13)	0.565
Lycopene (umol/L)	.09 (−1.9 to 3.7)	0.515	.08 (−7.6 to 13.4)	0.582	.08 (−60 to 101)	0.610
Zinc (ng/ml)	.15 (−.002 to .005)	0.396	.21(−.01 to .02)	0.211	- .23 (−.17 to .04)	0.209

* *P* value < 0.05

## Discussion

In this analysis, we found that low muscle mass was associated with poor mental health. However age, disability, chronic illness and tissue inflammation a marker of the severity of acute illness were also independently associated with poor cognitive function, increased depression symptoms and/or poor quality of life. We also found that patients with low muscle mass had poor nutritional biomarkers including B-group vitamins, antioxidants and trace elements.

Although poor muscle mass is known to be independently associated with increasing age and comorbidity and consequently leads to increased disability, morbidity and mortality information, on its effect on mental health and well-being is lacking [2, 3, 13]. Furthermore the contribution of poor nutritional biomarkers including B-group vitamins, antioxidants and trace elements in patients with low muscle mass to this poor outcome is also not known.

Possible candidates for underlying pathophysiological mechanisms linking changes in muscle mass and mental health include malnutrition, oxidative stress and inflammation [6]., First, there is direct and indirect evidence of the role of inflammation on loss of muscle mass in older people. In older patients inflammation associated with acute and chronic illness is known to lead to decreased food intake, increased nutrients demand and loss of muscle mass [14]. Inflammation induced cytokines and reactive oxygen species can directly mediate muscle damage resulting in accumulation of oxidative damage markers such protein carbonyls known to be independently associated with low grip strength [15, 16]. Several nutrients including those with antioxidant properties known to protect against inflammation were reported to be associated with muscle health [15–17]. Our results reveal low concentrations of a number of nutrients with antioxidant and anti-inflammatory properties in patients with low muscle mass. The ageing muscle for example, has been reported to show increased oxidative damage to DNA, protein and lipids. Antioxidant carotenoids quench free radicals and associated inflammation and therefore low concentrations have been found to be associated poor skeletal muscle function and disability [17]. Furthermore vitamin C a powerful antioxidant has also been shown to be significantly related to muscle strength in older women [18]. Treatment of old rats with a diet rich in some antioxidants and trace elements such was vitamins E and A, selenium, zinc and rutin has been reported to improve the stimulation of protein synthesis in muscles by the amino acid leucine. This effect may be mediated through reduction of inflammation and associated oxidative stress however the exact mechanism is yet to be determined [19].

Although many studies have reported a relationship between low muscle mass and physical outcome many of these studies have methodological concerns and few have addressed its relationship with mental health. For example, a cross-sectional community study of 3025 older women reported no significant association between low muscle mass and cognitive impairment after adjusting for confounders. However, the analyses suggest more severe stages of sarcopenia (low mass and/or function) could be associated with cognitive impairment [20]. Another small cross-sectional study reported significant associations between depressive symptoms, cognitive impairment and sarcopenia among healthy older men living in the community [21]. A community study from Belgium of older people aged 65 years or over reported an association between sarcopenia, cognitive impairment and quality of life. The authors however acknowledged a number of study limitations including the cross-sectional design, low power and the non-representativeness of the sample [22]. A relationship between poor self-reported general health and physical functioning was also reported in a very small community sample of subjects from England diagnosed with sarcopenia, compared with those without sarcopenia [23]. The sensitivity of generic scales used by most of the above studies to detect small effects of sacropenia on quality of life has questioned [24]. Work is ongoing to test the validity of a newly developed sarcopenia-specific quality of life questionnaire [24].

Physical inactivity in hospitalised older patients during both acute illness and recovery period is another possible reason linking muscle mass loss to poor mental health [5]. Exercise and skeletal muscles signalling has been linked to brain neurogenesis and cognitive functions however the extent to which this relationship is responsible for the beneficial effects of physical activity on mental well-being is not clear [5]. Physical activity confers benefit on most risk factors of ageing, but may also improve nutritional status by increasing energy expenditure, leading to increased nutrient intake if a mixed diet is consumed [25].

Some important limitations of this study include the number of exclusions and small numbers of patents with low muscle mass at follow up visits. Another important limitation is error known to be associated with measurement of anthropometric and biochemical nutritional indices in hospitalised older people. The longitudinal design and training and assessing observer’s error on anthropometric measurements, of the study and the use of a number of analyses to adjust for poor prognostic clinical indicators was to overcome some of these weaknesses.

The lack of direct correlations between blood borne micronutrients and mental health measures may partly be explained by small numbers. Nevertheless new evidence is emerging of a link between malnutrition and poor mental health and quality of life in older people and that improvement in nutritional status may leads to improvement in mental health and well-being [26]. There is an urgent need for more research in this field because the prospect of the effects of improved nutritional status of older people on mental health and quality of life could have an important and a substantial health and economic benefits.

## Conclusions

Low muscle mass in older patients is associated with poor nutritional status and mental health and well-being. Hitherto the evidence for the effectiveness of specific treatment or nutrient supplement is incomplete but sufficient to justify further research. Based on currently available evidence increased physical activity particularly following acute illness and a healthy diet with protein, fruits and vegetables to mitigate inflammatory responses is clearly appropriate for healthy independent ageing particularly for older patients.

## Data Availability

Data is available upon request to the corresponding author.

## Abbreviations

AMT: Abbreviated mental test questionnaire
EWGSOP: The European Working Group on Sarcopenia in Older people
GDS: Geriatric Depression questionnaire
SF-36: Medical Outcomes Study 36-items
